# Compassionate care through the eyes of patients and physicians: An interview study

**DOI:** 10.1371/journal.pone.0305007

**Published:** 2024-07-10

**Authors:** Maarten P. M. Debets, Iris Jansen, Mariëlle Diepeveen, Rosa Bogerd, Bert A. C. Molewijk, Guy A. M. Widdershoven, Kiki M. J. M. H. Lombarts

**Affiliations:** 1 Medical Psychology, Amsterdam UMC location University of Amsterdam, Amsterdam, the Netherlands; 2 Quality of Care, Amsterdam Public Health, Amsterdam, the Netherlands; 3 Ethics, Law and Humanities, Amsterdam UMC location Vrije Universiteit Amsterdam, Amsterdam, the Netherlands; University of Verona, ITALY

## Abstract

**Background:**

Although compassion is a crucial element of physicians’ professional performance and high-quality care, research shows it often remains an unmet need of patients. Understanding patients’ and physicians’ perspectives on compassionate care may provide insights that can be used to foster physicians’ ability to respond to patients’ compassion needs. Therefore, this study aims to understand how both patients and physicians experience the concept and practice of compassionate care.

**Methods:**

We conducted semi-structured interviews with eight patients and ten resident physicians at a University Medical Center in the Netherlands. Using thematic analysis, we separately coded patient and resident transcripts to identify themes capturing their experiences of compassionate care. This study was part of a larger project to develop an educational intervention to improve compassion in residents.

**Results:**

For both patients and residents, we identified four themes encompassing compassionate care: being there, empathizing, actions to relieve patients’ suffering, and connection. For residents, a fifth theme was professional fulfillment (resulting from compassionate care). Although patients and residents both emphasized the importance of compassionate care, patients did not always perceive the physician-patient encounter as compassionate. According to residents, high workloads and time pressures hindered their ability to provide compassionate care.

**Discussion and conclusion:**

Patients and residents have similar and varying understandings of compassionate care at the same time. Understanding these differences can aid compassion in medical practice. Based on the findings, three topics are suggested to improve compassion in residents: (1) train residents how to ask for patients’ compassion needs, (2) address residents’ limiting beliefs about the concept and practice of compassion, and (3) acknowledge the art and science of medicine cannot be separated.

## Background

Providing care with compassion is a crucial aspect of physicians’ professional performance and essential for achieving high-quality care [1]. Compassion involves the response to patients’ suffering coupled with the wish, intention, and action to relieve it [2, 3].‘Response to’ encompasses the reaction to the patients’ suffering, which in the case of compassion, is a virtuous response through relational understanding. That can sound like ‘I know you are suffering’. Action includes the act to relieve the suffering. For instance, by saying ‘what can I do to help you at this moment’. Research shows that compassion benefits patients’ clinical experience and alleviates their distress and anxiety [1, 3–7]. Moreover, compassionate patient care is associated with better patient self-care as it drives patient engagement and treatment adherence [1]. Although patients find compassion crucial, it is often an unmet need [1, 8–11]. In a national US telephone survey among 800 recently hospitalized patients and 510 physicians, only half of the patients reported receiving compassionate care [8]. Similar results have been found in Ireland and England, showing that patients reported the demonstration of compassion by the health care team fell short of what they considered important [12, 13]. The ramifications of this unmet need are serious, as less physician compassion has been associated with slower wound healing [5], less optimal blood sugar in diabetic patients [4], and higher levels of anxiety and pain [5]. Moreover, a lack of compassion is linked with lower quality of care, such as higher risks of medical errors [11].

While physicians agree with patients on the significance of compassion for successful medical treatment, they report that demanding work circumstances, such as excessive workloads and resulting time constraints, challenge their ability to provide compassionate care [8]. Moreover, during residency training, the skills and attitudes to provide compassionate care are not explicitly taught and role modelled [14]. Studies report decreased empathy–a crucial aspect of compassion–among residents and early physicians [15, 16]. This may endanger the quality of patient care and physicians’ well-being [1, 8, 11], as both self-compassion and compassion for others can mitigate burnout and increase physicians’ work engagement and job satisfaction [17, 18].

Healthcare institutions, and those working in them, are therefore expected to develop and sustain compassionate care in practice [13, 19–21]. However, evidence-based educational interventions equipping physicians with the knowledge, skills and attitude to provide compassionate care are mainly absent [21, 22]. This observation resonates with the conclusions of Blomberg’s et al. systematic review about interventions for compassionate nursing care: “While there have been many published studies that appear to offer potential solutions to deficits in compassionate care, this is a body of literature that seems to have little useful to say to nurses in practice.” (p.153) [23]. Internationally, researchers have started to explore physician behaviors that patients experience as compassionate [1, 24, 25]. Knowing how patients and physicians experience compassionate care is required to best serve patients, to teach compassionate care and to design improvement interventions. In contrast to the nursing profession [26–28], the focus on physicians’ experiences of compassion in clinical practice remains even more limited [29], and the voice of patients, needs further exploration [10, 26]. The few studies that touched upon patients’ experiences of compassion predominantly focused on cancer patients. These studies showed that compassion for these patients included the health care provider’s understanding of patients’ needs, relational communicating, and attending to needs [10, 30].

Against this background, this study aims to understand patients’ and physicians’ experiences of compassionate care by identifying key themes for both. Such an understanding can serve as a basis for educational interventions aimed at fostering compassionate care by physicians, by providing insights into the differences between views of patients and physicians on compassion and into potential barriers for compassionate care. Indeed, missed opportunities to provide compassionate care represent a significant challenge [21, 31, 32]: when physicians are unaware of what compassionate care means for patients, providing compassionate care is complicated. Therefore, this study answers the following research question: What are experiences of patients and physicians regarding compassionate care?

## Methods

This interview study was conducted among patients and resident physicians (hereafter: residents) at a large University Medical Center (UMC) in the Netherlands. Residents are physicians in training and are responsible for providing patient care. This study was part of a larger project to develop an educational intervention to improve compassion in residents.

### Research team and reflexivity

Given the qualitative nature of the study all researchers actively engaged in discussions, bringing their own backgrounds to the analytical process. The research team consisted of researchers from various disciplines; all are familiar with physicians’ professional performance and compassionate care. MD is a social scientist with a background in Strategic Human Resource Management (MD); IJ is a sociologist with a PhD in medical education; MDi is a PhD student and lecturer medical ethics with a background in medicine; RB is a social scientist with a background in bioethics and public administration; BM is a clinical ethicist and social scientist; GM is philosopher and qualitative social scientist with large experience in ethics and research; and KL is a health services researcher with over 30 years of experience in working with medical professionals. The research teams’ multifaceted perspectives resulted in in-depth conversations on compassionate care and the patient-physician interaction.

### Sampling and data collection

Patients and residents at the UMC were selected through convenience sampling. Although this meant patients and residents who were willing to participate were interviewed, for recruiting patients and residents we strived for diversity in terms of age, education, and year of medical education. We recruited patients via medical specialists and the Client Advisory Council in the UMC. Residents were recruited via Program Directors and through snowballing by asking participating residents. All participants were invited by phone call or e-mail, and they received a study information letter by e-mail.

The research team developed semi-structured interview guides for patients and residents. The guides contained the same questions; only the wording was tailored to patients or residents (S1 Appendix). During the interview, patients and residents were asked to describe their experiences with respectively receiving and providing compassionate care to explore their perceptions. Other main interview topics included perceptions of too much or too little compassion, barriers and facilitators of compassionate care, and attention for compassion in residents’ working environment. All interviews were conducted between August 2019 and December 2019 by authors MD, IJ, RB, and MDi. Patient interviews were conducted within the UMC or at a preferred location of the patient, e.g., at their house. All resident interviews were conducted within the UMC. The interviews for patients and residents lasted between 40 and 75 minutes; 60 minutes on average. After conducting the interviews, the researchers discussed the interview and shared reflections as it facilitated the following interviews. Interviews were audiotaped, transcribed verbatim, and anonymized before data analysis.

### Ethical approval and consent to participate

The institutional ethical review board of the Amsterdam UMC of the University of Amsterdam provided a waiver declaring the Medical Research Involving Human Subjects Act (WMO) did not apply for the project (reference number W19_327 # 19.386). All participants provided written informed consent. Participation in the study was voluntary at all times.

### Data analysis

All interviews were analyzed using template analysis, which is a form of thematic analysis [31]. We followed the six main procedural steps for conducting template analysis as outlined by Brooks et al [31] and King [33]. After becoming familiar with the data (step 1), three resident transcripts were read and open coded independently by MD and IJ (step 2). Then, MD and IJ discussed the codes and constructed themes (i.e., how codes cluster together) (step 3), resulting in an initial template (step 4). After the research team discussed the template, IJ applied the template to the following transcripts. We refined the themes iteratively during regular team meetings until we agreed upon the template (step 5). In the final stage, IJ applied the template to all transcripts (step 6). Then, the same data analysis steps were applied to patient interviews, i.e., a separate template was developed for patients. We regularly checked throughout the interviews whether patients were talking about physicians or other healthcare professionals; when possible the latter fragments were excluded. After analyzing ten resident and eight patient transcripts, saturation was met, meaning we had collected enough data to globally understand how patients and residents experienced compassionate care and where differences occurred. MAXQDA (version MAXQDA Plus 2020) supported data analysis.

## Results

In total, eight patients (5 female) participated in an interview. Patients were between the age of 22 and 79. Patients were under treatment within the (sub)specialties of cardiology (4) or internal medicine (4). Ten residents (8 female) participated in an interview. They varied in postgraduate years (ranging from year 1 to 6) and specialty, including radiology (1), internal medicine (5), ophthalmology (1), and surgery (3).

The data analysis identified four interrelated themes that express what compassionate care entails for patients and residents: (1) being there, (2) empathizing with the patient’s suffering, (3) actions aimed to relieve this suffering, and (4) connection. Within these four themes, different subthemes emerged for patients and residents. For residents, a fifth theme was professional fulfillment resulting from compassionate care (see Table 1). Generally, patients and residents emphasized the importance of compassion for optimal patient care. Nevertheless, patients also shared that they did not always perceive their care as compassionate, and residents touched upon the challenges in providing compassionate care to their patients.

**Table 1:** Themes and subthemes for patients and residents expressing compassionate care.

Theme	Subthemes of patients	Subthemes of residents
**Being there with and for patients**	Displaying attention by taking time and being prepared (+)	Taking the time for patients (+)
	Having a demeanor of calmness (+)	Taking responsibility to manage patients’ healthcare processes (+)
	Showing stress (-)	
**Empathizing**	Seeing and treating patients as a person (+)	Seeing patients as fellow human beings (+)
	Asking about patients’ needs (+)	Standing in patients’ shoes (+)
	A lack of empathy (-)	Balancing over-involvement and detachment (+/-)
		Judging the severity of patients’ medical condition (+/-)
**Action**	Communicating clearly (+)	Small and extraordinary actions (+)
	Involving patients during the medical process (+/-)	Relieving patients’ suffering (+)
		Time pressures and lack of time (-)
**Connection**	To click with someone (+)	To click with someone (+)
	Equal relationship (+)	Reciprocal relationship (+/-)
	Engaged and medically interested patients (+)	Bad mood (-)
**Professional fulfillment**	N.A.	Making a difference for patients (+)
	N.A.	Organizational hassle (-)

(+) indicates that the subtheme had a positive influence on compassion.

(-) indicates that the subtheme had a negative influence on compassion.

## Patients

### Being there with and for patients

Patients perceived physicians’ attention when they literally took (more) time by, for example, planning them “at the end of the consultation so that I had enough time to ask questions” (P3). When physicians had well prepared for the consultation and were informed about their situation, patients felt that physicians were interested in them.

Also, patients experienced physicians being there for them through their demeanor of calmness by sitting down while talking to patients as it “shows that [physicians] have more time instead of just quickly call on me” (P5). In contrast, when physicians were stressed or in a hurry, patients felt being a “box that should be ticked off” (P5). In these situations, they felt unheard, less free to ask questions and, at times, a burden.

### Empathizing

Patients felt being seen as a person when physicians were understanding and caring, rather than only “examining the heart and moving on” (P5). According to patients, empathy could be expressed by saying “I notice what it does to you, it is intense” (P5).

They frequently indicated that physicians should ask patients “what do you need” (P4). This subtheme was closely related to the previous one, as asking contributes to being seen as a person. When physicians did ask such questions, patients felt taken seriously, and it was easier for them to open up and share thoughts or concerns:

‘What do you need?’. Who is that person sitting across from me? […] I really believe that then you will have a better conversation […] seeing the complete picture and not only the illness sitting across from you, really seeing the person facing you.

(P3)

Patients also provided narratives showing how they experienced physicians’ lack of empathy. They felt that physicians did not always recognize their unease or behaved inappropriately, which evoked emotional reactions:

Then he puts his ultrasound on [belly pregnant patient]. He says ‘O, I can only see four fingers and a thumb’, which was meant as a joke. I thought, well, you have no idea how I feel, as it was the special clinic for high-risk pregnancies. […] I was terrified because I thought, now it is all wrong.

(P5)

### Action

Patients recognized the relevance of action as an element of compassion: physicians doing something to relieve their suffering, in which suffering could be for example pain, confusion or uncertainty. An act of compassion could be remembering what patients had said in an earlier consult or comforting patients by saying “it’s going to be okay” (P4). Patients indicated two specific actions that could relieve their suffering: communicating clearly, and involving patients in the medical process.

For patients, clear communication included physicians explaining a medical procedure step-by-step. Patients found it important that physicians were aware of their way of communicating as “c’est le ton qui fait la musique [it is the tone that makes the music]” (P8) and that physicians adjusted their communication to their understanding. The importance patients ascribed to clear communication became apparent as patients highlighted how medical interventions could technically be successful but fail in the sense that patients still suffered from confusion and uncertainty due to insufficient explanation.

For patients, action also included involving them in the medical process, enabling them to maintain voice and control. When physicians involved patients, for example, during decision-making processes, they felt taken seriously. In contrast, non-involvement could evoke emotions such as frustration or anger. Especially during physical examinations, patients felt extra vulnerable and were upset when physicians did not involve them in what was going to happen or did not provide reassurance:

Well, I was lying there in the room covered by my towel, so that makes you vulnerable, right. And he walked in […] Immediately, a blob on the ultrasounds head, pulling down the towel and putting the ultrasound on my skin. It’s really an invasion of your body.

(P2)

### Connection

Patients described a connection with a physician as to “click with someone” which could promote a long-term relationship with the physician:

I have been a patient with [physician] for six years. […] I noticed that we very quickly developed a certain mutual sympathy. We have the same kind of humor, I think. And the connection was there right away. […] We have a connection, understand each other and you do not have to make that explicit.

(P8)

For patients, a connection with physicians also entailed a relationship based on equality, meaning physicians are symbolically “standing next to me, instead of positioning themselves above me” (P4). As a result, patients noticed being more forgiving (e.g., if a consultation runs late), taking physicians’ advice more seriously, and feeling safer to ask questions. Finally, patients noticed physicians’ enthusiasm when patients were engaged and interested in their own medical situation through, for instance, preparing the consultation and asking questions.

## Residents

### Being there with and for patients

By taking time for patients, residents intended to give them the feeling that they were being heard and taken seriously. Removing distracters (e.g., pagers) and sitting down with patients helped residents to take time for patients and be present.

Also, residents talked about the responsibility to manage healthcare processes, such as making phone calls or consulting other physicians. Even when residents could not physically be there for patients, i.e., because their shift had ended or they were on leave, they still felt responsible for searching for replacement:

If a new patient shows up at four o’clock, you can’t say I only work until five. You have to find at least someone else to see the patient. […] Being a physician is not just an activity, rather it is a responsibility.

(R3)

### Empathizing with patients

For residents, empathy included looking beyond the patients’ disease and seeing them as fellow human beings by, for example, asking patients about:

Hobbies and the home situation. If you just ask people, what do you like to do? Then a patient changes into a human being.

(R4)

Residents empathized with patients by metaphorically standing in their shoes: residents imagined how they themselves would like to be treated or how “I would like my mother to be taken care of” (R9). While residents described the ability to empathize as a personality trait, they also stressed that it can be learned and developed. Empathizing with patients could also create tensions for residents, for example when trying to find the balance between over-involvement and detachment in patient encounters:

You can’t cry along with every patient. Then you can’t practice medicine because tremendously miserable things happen.

(R8)

Empathizing was facilitated or hindered by residents’ judgment of the severity of patients’ medical condition. When residents judged the medical situation as severe, feelings of empathy arose and providing compassionate care was “more obvious and recognizable” (R6). On the other hand, when residents evaluated the situation as less severe, being compassionate felt less necessary, e.g., when patients had undergone the treatment before and knew what to expect.

### Action

While residents highlighted that actions could be small gestures (e.g., putting a hand on a shoulder, naming and recognizing the suffering) which are “the little things one can do” (R9), they also often talked about out-of-routine care efforts, such as visiting the patient once more during on-call shifts, or extraordinary action.

Regardless of whether the actions were small or extraordinary, residents aimed to relieve patients’ suffering. For example, one resident described alleviating patients’ stress by playing their favorite music during surgery:

So, in that sense, it just created peace for him. That’s a good thing, of course. Because it means that the anesthesia team–being busy with the epidural–can focus on that while the man [patient] experiences less stress. And of course, you can clearly observe that in the heart rate. You can see that very nicely when someone is attached to the monitor in the OR.

(R9).

Residents consistently reported how a lack of time and time pressures constrained their ability to act compassionately. Residents experienced “too little time per patient” (R1) and felt unable to go that “extra mile and perhaps listen a little more [to the patient]” (R1), as their outpatient clinic would run late. Moreover, as some residents experienced high time pressures during consults with patients, they noticed that being compassionate “sometimes slips through the cracks” (R10).

### Connection

In line with patients, residents described a connection as to “click with someone”. They noticed that a connection with patients could be fostered by identifying with patients, being moved by their stories, or being involved in patients’ care process for an extended period. Moreover, some residents felt that a connection was promoted when patients expressed interest in medicine, e.g., by describing “very clear symptoms” (R5).

Residents saw a reciprocal relationship as important, meaning that patients behaved appropriately since the patient-resident relationship is a matter of “giving and taking” (R10). Hence, providing compassionate care felt more natural when patients were polite, respectful, and were interested in residents. In contrast, patients who residents perceived as demanding or complaining challenged residents’ ability to express compassion. Residents were also challenged by patients that expressed ideas that “go against your convictions” (R2). One resident asked herself whether her annoyance towards a patient could have affected her medical judgment:

And if you compare it with patients who have metastasized cancer, then they [patients with breast cancer] nag too easily really, that is the kind of the feeling you get. […] So did this lady [patient]. […] There was not much of a chance that something was wrong. And then, later, it turned out that she had metastases everywhere. […] I wondered whether my lack of patience or empathy actually hampered my assessment

(R4).

Residents also recognized how being in a bad mood could interfere with establishing and maintaining a connection. If they, for example, “had a terrible night of sleep and were cranky” (R6), residents had the feeling that they provided less compassionate care and found themselves “less nice to patients” (R6).

### Professional fulfillment

As a result of providing compassionate care, residents reported that they felt “much more fulfilled as a physician” (R7). While residents generally felt satisfied by providing patient care, being compassionate entailed the feeling of really making a difference for patients. This was especially experienced in situations where patients expressed their contentedness as a result of the resident guiding them through important health events in their live:

Sometimes I even get a few goosebumps […] and I think what a unique profession we have, […] that we are allowed to guide people in this. […] Of course it also takes energy because you discuss heavy themes, but the fact that you can help people so well. Sometimes I walk out of the consulting room: wow, special.

(R2)

## Discussion

This study sought to understand how patients and residents experience compassionate care and identified four themes for patients and residents: (1) being there, (2) empathizing with the patient’s suffering, (3) actions aimed to relieve this suffering, and (4) connection. For residents, a fifth theme was professional fulfillment resulting from compassionate care. The identified themes resonate with previous research on compassion [10, 29, 30, 32] and add to a more comprehensive understanding of compassion in the physician-patient encounter. Interestingly, in a virtual care context patients identified similar key elements of compassionate care such as ‘holistic care’ (“care that extends beyond the presenting symptom(s) to include the patient context and broader needs”) and encounter-based presence (“feeling prioritized by and connected to the clinician during the visit”) [24].These finding illustrate the importance of training physicians to provide compassionate care both in physical and virtual settings. An important finding of this study is that patients did not always perceive compassion in the physician-patient encounter. At the same time, all residents spoke of their intentions to provide compassionate care. Thus, the findings indicate a gap exists between patients’ experiences and residents’ intentions. More closely inspecting the identified themes provides insight into the shared and unique experiences of patients and residents regarding compassion, which may inform educational interventions to bridge this gap.

The additional theme for residents, professional fulfillment, confirms that physicians are mainly driven by their desire to help patients [34]. Also, this finding suggests that enhancing compassion in residents will likely benefit the quality of patient care and residents’ well-being. For most of the shared themes, there were only subtle differences in patients’ and residents’ experiences of compassion. Within the action theme, there was a more notable difference. In comparison to patients, residents more often experienced providing compassionate care as performing additional (medical) actions. A closer inspection of the themes also shows that residents judged patients’ need for compassion based on the severity of their medical condition, and some described providing compassion when they felt they had enough time. These findings suggest residents hold several limiting beliefs (e.g., it takes too much time) about the concept and practice of compassion.

Based on the findings, we will now discuss three topics that need attention in order to improve compassion in residents: (1) train residents how to ask for patients’ compassion needs, (2) address residents’ limiting beliefs about the concept and practice of compassion, and (3) acknowledge the art and science of medicine cannot be separated.

### 1. Train residents’ how to ask for patients’ compassion needs

For patients, small gestures were experienced as an act of compassion, such as a physician putting a comforting hand on the patient’s shoulder. This insight may bring compassionate care within the reach of more patients without much extra costs or ‘hard work’, when residents start noticing and responding to patients’ specific, attainable compassion needs. In line with previous findings [2, 29, 30], residents often perceived that assessing patients’ compassion needs comes down to the adage ‘do unto others as you would have them do unto you’. However, from patients in this study, it can be learned that this approach may not always accurately reflect how patients wish to be treated. Patients suggest that healthcare providers could ask them about their needs instead of making assumptions about their needs. It should be noted here that using such questions (e.g., ‘what do you need’) merely instrumental, without making a meaningful and sincere connection with patients, is likely to be less effective [1]. Studies on physician-patient communication provide guidelines on how to ask patients for their care preferences, which may inform educational interventions to indeed make a meaningful connection and identify patients’ compassion needs [35, 36].

### 2. Address residents’ limiting beliefs about the concept and practice of compassion

This study suggests that residents hold several limiting beliefs about the concept and practice of compassion. Residents occasionally experienced providing compassionate care as difficult, hard work, and an increase to their workload. They experienced providing compassionate care as time-consuming, especially when their clinics ran late. Previous studies show that demanding working circumstances constrain residents’ ability to provide compassionate care [8, 17]. However, the perception that compassion includes ‘extra’ work and cannot be practiced in challenging work environments may entail a limiting belief. Researchers found that small gestures and compassionate communication would only take less than forty seconds per patient and reduces patients’ anxiety levels [37, 38]. For example, using the strategies of “confirmation”–conveying that the patient’s emotion or concern is legitimate–or “pursuit”–asking the patient a follow-up question or offering support took on average about half a minute [1]. Also, compassionate messages from oncologists at the beginning of the consultation i.e., “[…] we are in here together and we will go through this together” and at the end of the consultation i.e., “[…] I will be with you each step along the way” took forty seconds and reduced patients’ anxiety levels [37]. Patients in this study particularly experienced physicians’ humane qualities and behavior, such as treating patients as equals by sitting beside them, as valuable.

Residents also found it challenging to determine the appropriate level of compassion, as they were afraid to become too involved and emotionally attached. This may represent a limiting belief because when compassion is understood and practiced appropriately, it also yields a range of positive outcomes and may even reduce emotional exhaustion [17, 22, 39, 40]. Therefore, additional attention is needed to train residents to determine an appropriate and balanced position regarding compassion. Virtue ethics focuses on the disposition which enables one to find the right middle between two extremes [41–43]. Educational interventions may include insights from virtue ethics and previously noted evidence on the effects of compassion which can enable residents to reflect upon how to determine their appropriate, balanced position on a compassion continuum, namely finding their position between detachment and over-involvement [43, 44].

Lastly, residents found it challenging to connect with certain patients, hampering their ability to provide compassionate care. For example, when residents encountered patients that, in their eyes, misbehaved or had undefinable medical complaints. The opposite was also true; when residents identified with patients or were interested in their medical condition, it facilitated humanistic practice, resonating with previous findings [45–47]. While identification with certain people and personalities is inherent in all human relationships, a positive physician-patient connection is crucial for patients’ health and should therefore be established regardless of patients’ characteristics [1]. Despite the best intentions of physicians, implicit and explicit biases, for example, regarding race, culture, gender, or the patient’s medical condition, influence the patient-physician encounter and patient outcomes [48, 49]. Hence, in an educational intervention, residents can explore their prejudices as awareness of them may help to attenuate instinctive uncompassionate actions regarding challenging patients [50].

### 3. Acknowledge the art and science of medicine cannot be separated

Our study confirms that compassion is an essential aspect of medical care. Internationally, the importance of compassion is emphasized in, for example, the American Medical Association’s Principles of Medical Ethics and the British NHS constitution [32]. However, our results suggest that some residents believe high-quality care can exist without compassion. While this can also be seen as a belief limiting residents’ compassion efforts, this topic deserves more attention in medical practice in general. Implicit messages within the medical environment champion certainty, objectivity, and physicians’ procedural skills, while the more elusive qualities like communication and compassion are rarely explicitly included in residency training programs [14, 51, 52]. Leaders in healthcare, supervisors, and training programs must acknowledge that the art and science of medicine cannot be separated and appreciate and value both aspects accordingly, for instance, by supervisors being role models and providing explicit feedback on compassion to residents.

### Strengths and limitation

The strengths of this study are the inclusion of both patients and residents and explicitly comparing these insights to elaborate directions for developing educational interventions to improve compassion in physicians, thereby addressing a shortage in the literature [17, 21, 22]. A limitation of this study is that the participating residents were mainly white women, which mirrors the Dutch resident population but likely influenced our results as providing compassionate care might be shaped by such characteristics [17]. For example, researchers found that female physicians’ practice patterns included more patient-centered, empathetic communication [52]. Moreover, most participating patients were well-educated, white women. Also, patients’ different medical histories may have influenced their perspectives on compassion which could further limit the transferability of this study’s findings.

## Conclusions

For both patients and residents, we found that compassionate care encompassed: being there, empathizing, actions to relieve patients’ suffering, and connection. For residents, compassion included a fifth theme: professional fulfillment. Patients and residents emphasized the importance of compassion, although they had different views about compassionate actions. According to residents, compassionate actions often included ‘doing more’ in terms of (medical) actions, while patients especially valued residents’ human qualities of ‘being with’ the patient and/or making a human-to-human connection with the patient. Residents’ ability to provide compassionate care depended on their perceptions (e.g., it takes too much time), patients’ characteristics, and their workload and available time. To improve compassion in residents our findings suggest to (1) train residents how to ask for patients’ compassion needs, (2) address residents’ limiting beliefs about the concept and practice of compassion, and (3) acknowledge the art and science of medicine cannot be separated.

## Supporting information

S1 Appendix (DOCX)
