# Supplementary material for: Compassionate care through the eyes of patients and physicians: An interview study
Source: PLoS One. 2024 Jul 10;19(7):e0305007. doi: 10.1371/journal.pone.0305007 (PMC11236150; doi:10.1371/journal.pone.0305007)
Supplement: S1 Appendix — (DOCX) [file pone.0305007.s001.docx]

**Additional file 1: Semi-structured initial interview guide for residents and patients**

Not all follow-up and probing questions are included in the interview guides below. During interviews with patients, we regularly checked whether patients talked compassionate care provided by residents.

­­­­­­­­­­­­­­­­­­­­­­­­­­_________________________________________________________________________________

Interview guide residents

*Introductory questions*

1. Can you please indicate, in three to five words, on this paper what associations compassion evokes in you?
2. What is the value of compassionate care for you?

*Key questions*

1. Can you give an example of a situation in which you felt successful in providing compassionate care?
2. Can you also give an example of a situation in which you felt less successful in providing compassionate care?
3. What would be too much or too little compassion for you?
4. What helps you in providing compassionate care to patients?
5. What hinders you in providing compassionate care to patients?
6. Within your current work environment, is there attention for compassion?
7. How good are you at providing compassionate care? Does it feel like a second nature, or do you have to put effort into it?

*Wrap-up questions*

1. Are there topics that I did not ask about but are essential?

Interview guide patients

*Introductory questions*

1. Can you please indicate in three to five words on this paper what associations compassion evokes in you?
2. How did you experience compassion in your health care trajectory within this hospital?

*Key questions*

1. Can you give an example of a situation where you felt that your physician provided compassionate care?
2. Can you also give an example of a situation where you felt that your physician provided less compassionate care?
3. Looking back at the examples you just offered, how do you feel when you:
   1. Are treated compassionately by your physician?
   2. Are treated less compassionately by your physician?
4. What would be too much or too little compassion for you?
5. What aspects contribute to your experience of compassionate care?
   1. What factors promote the experience?
   2. What factors hinder the experience?
6. What tip would you give to physicians so that they can provide (more) compassionate care?

*Wrap-up questions*

1. Are there topics that I did not ask about but are essential?
